# Long-term blood pressure outcomes of laparoscopic adrenalectomy in trHTN patients

**DOI:** 10.2478/jtim-2021-0005

**Published:** 2021-01-05

**Authors:** Yue Deng, Hanbo Wang, Xudong Guo, Shaobo Jiang, Jun Cai

**Affiliations:** Hypertension Center of Fuwai Hospital, State Key Laboratory of Cardiovascular Disease, National Center for Cardiovascular Diseases, Chinese Academy of Medical Sciences and Peking Union Medical College, Beijing 100037, China; Department of Urology, Shandong Provincial Hospital affiliated to Shandong First Medical University, Jinan 271016, Shandong Province, China

**Keywords:** treatment resistant hypertension, adrenalectomy, blood pressure, surgery

## Abstract

**Background and Objectives:**

Treatment resistant hypertension (trHTN) is a common clinical problem faced by many clinicians. Laparoscopic adrenalectomy effectively trims blood pressure (BP) elevation secondary to various functional adrenal disorders. However, the impact of adrenalectomy on BP within trHTN patients has never been reported. Our present study aims to investigate the effect of adrenalectomy on BP management within trHTN patients, and to explore clinical predictors for postoperative BP normalization.

**Patients and Methods:**

In our current study, 117 patients diagnosed with trHTN and performed with unilateral adrenalectomy were consecutively enrolled, demographic and medical information were documented for baseline data collection. BP was measured with a standard electronic sphygmomanometer twice a day. Long-term periodical interview was conducted and 109 (93.2%) enrolled patients were successfully followed-up at an averaged 36.2 months.

**Results:**

At follow-up, 27/109 (25%) trHTN patients acquired BP normalization and 68/109 (62%) patients acquired BP improvement. Mean taking anti-hypertensive agents reduced from presurgical 4.24 to present 1.21 (*P* < 0.01), along with 7.2 mmHg reduction in SBP (*P* < 0.01). Image macro-adenoma and hypokalemia history were found to be the two strongest predictors for postoperative BP normalization. (*χ^2^*= 28.032, *P* < 0.01). The incidence of adverse postoperative events was quite small.

**Conclusions:**

In summary, this current study implicates that adrenalectomy is an efficacious and safe surgical strategy for BP management in trHTN patients. Patients with both unilateral macro-adenoma and hypokalemia are more prone to acquire postoperative BP normalization.

## Introduction

Treatment resistant hypertension (trHTN) is a common clinical problem faced by both primary care clinicians and specialists, defined as the failure to achieve target blood pressure (BP) despite on optimal doses of an appropriate triple antihypertensive regimes, a diuretic included,^[1]^ or controlled hypertension on at least 4 drugs.^[2]^ In addition to its relatively high prevalence, occurring in estimated 10%–20% of total treated hypertensive patients,^[3]^ trHTN patients also have an approximately 1.5–3 fold increased risk of cardiovascular events and mortality compared to patients with more easily controlled hypertension, which cause a huge burden to both individuals and the whole society.^[4, 5, 6]^ To solve this tricky clinical puzzle, several surgical interventions including renal denervation, deep brain stimulation and carotid baroreceptors’ stimulation have been recently developed for the management of trHTN. However, none has been widely employed since the related clinical trials and evidence are still insufficient or inconsistent.^[7, 8, 9]^

A sizable number of trHTN patients may have adrenal lesions.^[10,11]^ Hormone overproduction, next to malignance is the major concern of this problem. Diverse functional adrenal lesions including aldosterone-producing adenoma (APA),^[12]^ adrenal Cushing's syndrome^[13]^ and pheochromocytoma,^[14]^ excessively produce a series of adrenal hormones to elevate BP and even deteriorate cardiovascular functions directly. Intriguingly, recent studies have implied that nonfunctional adrenal tumors (NFATs) also links with higher incidence of diabetes,^[15]^ hypertension and cardiovascular events as well.^[16]^ Laparoscopic adrenalectomy is demonstrated as an effective and safe strategy for correcting excessive adrenal hormones-induced secondary BP elevation and deleterious cardiovascular impact, and has been recommended as the first choice of treatment in most functional adrenal disorders. Moreover, hypertension resolving after adrenalectomy for NFATs was observed in several studies as well.

Clarifying the effect of laparoscopic adrenalectomy on BP targeted in trHTN patients may be instructing for BP management within this population, whereas no study has reported this. Thus, our present study is aimed at reporting the long-term BP outcomes of laparoscopic adrenalectomy in trHTN patients and further interrogating into the clinical indicators predicting for postoperative BP normalization.

## Materials and Methods

### Subjects selection criteria

From January 1^st^ 2011 to July 1^st^ 2017, patients hospitalized in Shandong Provincial Hospital, who met the following 3 criteria, were consecutively enrolled into our study: (1) Systolic blood pressure of 140 mmHg or more despite being treated with at least 3 antihypertensive drugs, 1 diuretic included, or controlled hypertension on at least 4 drugs; (2) Partial or total adrenalectomy was performed on one side suspicious adrenal gland; (3) Inconclusive biochemical diagnosis of adrenal lesions. Of note, hypertensive patients with adrenal lesions are supposed to be performed with elaborate endocrine examinations to screen for functional adrenal disorders according to the guidelines.^[17,18]^ However, complete discontinuation of all interfering drug within our participants was confined by cardiovascular safety concerns. Thus, hormone examination results of our participants were not taken into consideration and adrenal lesions was biochemically diagnosed as “inconclusive”. This study was conducted in accordance with the Declaration of Helsinki and approved by the ethnic commission of Shandong provincial hospital, and information consents from each participant were acquired.

### Data collection and BP measurement

Collected data included demographic information, preoperative BP, anti-hypertensive medication regimes, computed image scan findings and postoperative histopathological reports of adrenal lesions. BP was measured twice per day at 7 am and 15 pm after a 10-minute rest at 3-minute intervals with standard electronic sphygmomanometer,^[19]^ the average of BP values measured within consecutive 4–5 days before surgery was calculated and recorded. All the patients underwent preoperative adrenal computed image with computed tomography (CT) or magnetic resonance (MR). The presentations on image were divided into four groups: (1) Unilateral macro-adenoma, which was defined as an existence of cortical macro-adenoma with a normal contralateral gland on image,^[20]^ while macro-adenoma was defined as the presence of a definite low-dense homogenous mass with clear boundary and diameter more than 10 mm on adrenal image scans; (2) Unilateral combined lesions (the coexistence of both macro-adenoma and adrenal hyperplasia on ipsilateral adrenal gland); (3) Unilateral adrenal hyperplasia in combination with or without micro-adenoma (diameter less than 10 mm on adrenal image scans); (4) Bilateral adrenal lesions. In our current study, unilateral macro-adenoma and unilateral combined lesions are collectively referred to as “image macro-adenoma”.

### Laparoscopic adrenalectomy

All the patients in this study chose to proceed with surgically treatment, due to certain incompatible complications. TrHTN, consistent hypokalemia, early onset stroke or kidney dysfunction were the main drivers. In the current study, retroperitoneoscopic procedures were the standard treatment modality for adrenal lesions and were performed by experienced surgical team in Shandong Provincial Hospital. Total adrenalectomy was performed in 106/117 (90.6%) patients and subtotal adrenalectomy was performed in other 11 patients. Histopathologic reports of surgical specimen from all the patients were obtained.

### Follow-up information

To determine the effect of adrenalectomy, patients were asked to measure BP with aforementioned standard electronic sphygmomanometer by themselves twice a day, and invited with regular follow-up visits to check for general physical condition and laboratory examinations if necessary. During July 2017 to October 2017, we contacted the patients via telephone interview to get the finally updated information about their BP, anti-hypertensive medications, and where ever possible, plasma potassium level. We studied the group of patients who had follow-up data to ascertain factors correlated with BP prognosis after adrenal surgery.

### Assessment of outcomes

Postoperative BP outcomes were allocated into three groups based on the final interview: (1) normalization: normotension (less than 140/90 mmHg) with total cessation of antihypertensive agents; (2) improvement: lower BP levels on similar number of or lower dose of antihypertensive agents; (3) non-response: unaffected BP level on similar or more antihypertensive agents. Hypokalemia resolution was defined as normal potassium level without potassium supplementation.

### Statistical analyses

Quantitative variables with normal distribution are present as mean and standard deviation. Categorical variables are reported as frequencies. Associations of quantitative parameters with outcomes were evaluated by means comparison employing the unpaired t test or ANOVA test, while association of categorical variables with outcome was evaluated with the *χ^2^* test, or Fisher exact test if appropriate. Logistic regression was used to provide an assessment of which variables made an independent contribution to probability of surgical cure. Bivariate correlation analysis was conducted among the factors with evident association with BP prognosis. *P* < 0.05 was considered statistically significant. SPSS 21.0 software was used for statistical analyses in our study.

## results

### Baseline demographic and clinical characteristics of participants

From January 1^st^, 2010 to July 1^st^, 2017, 117 patients who met all three aforementioned inclusion criteria were consecutively enrolled in our study. Table 1 summarizes the baseline characteristics of the initial 117 participants. The average age of patients was 47.5 years and 35% (*n* = 41) were female. Preoperative BP was 143/87 mmHg on an averaged 4.21 antihypertensive agents. According to the image scan findings, these 117 patients was diagnosed as: 36 (31%) with unilateral macro-adenoma, 19 (16%) with unilateral combined lesions, 30 (26%) with unilateral adrenal hyperplasia and 32 (27%) cases with bilateral adrenal lesions. Surgical specimen histopathological reports from all the participants were also consistent with the subgrouping according to image screening.

**Table 1:** Baseline characteristics of study participants

Item	
Female	41 (35 %)
Age (year)	47.5±12.3
Hypertension Onset Age (year)	38.2±10.6
Hypertension Duration (year)	9.5±8.4
SBP (mmHg)	142.5±11.9
DBP (mmHg)	86.5±9.2
Antihypertensive drug numbers	4.21±1.06
Plasma Potassium (mmol/L)	3.39±0.97
Hypokalemia	52 (44%)
Diabetes	31 (26%)
Stroke	25 (21%)
Surgical options (total)	106 (91%)
Image subtypes	
Unilateral macro-adenoma	36 (31%)
Unilateral combined lesions	19 (16%)
Unilateral adrenal hyperplasia	30 (26%)
Bilateral adrenal lesions	32 (27%)

SBP: systolic blood pressure; DBP: diastolic blood pressure.

### Adrenalectomy represented an effective strategy for BP control in trHTN patients

**Figure 1:**
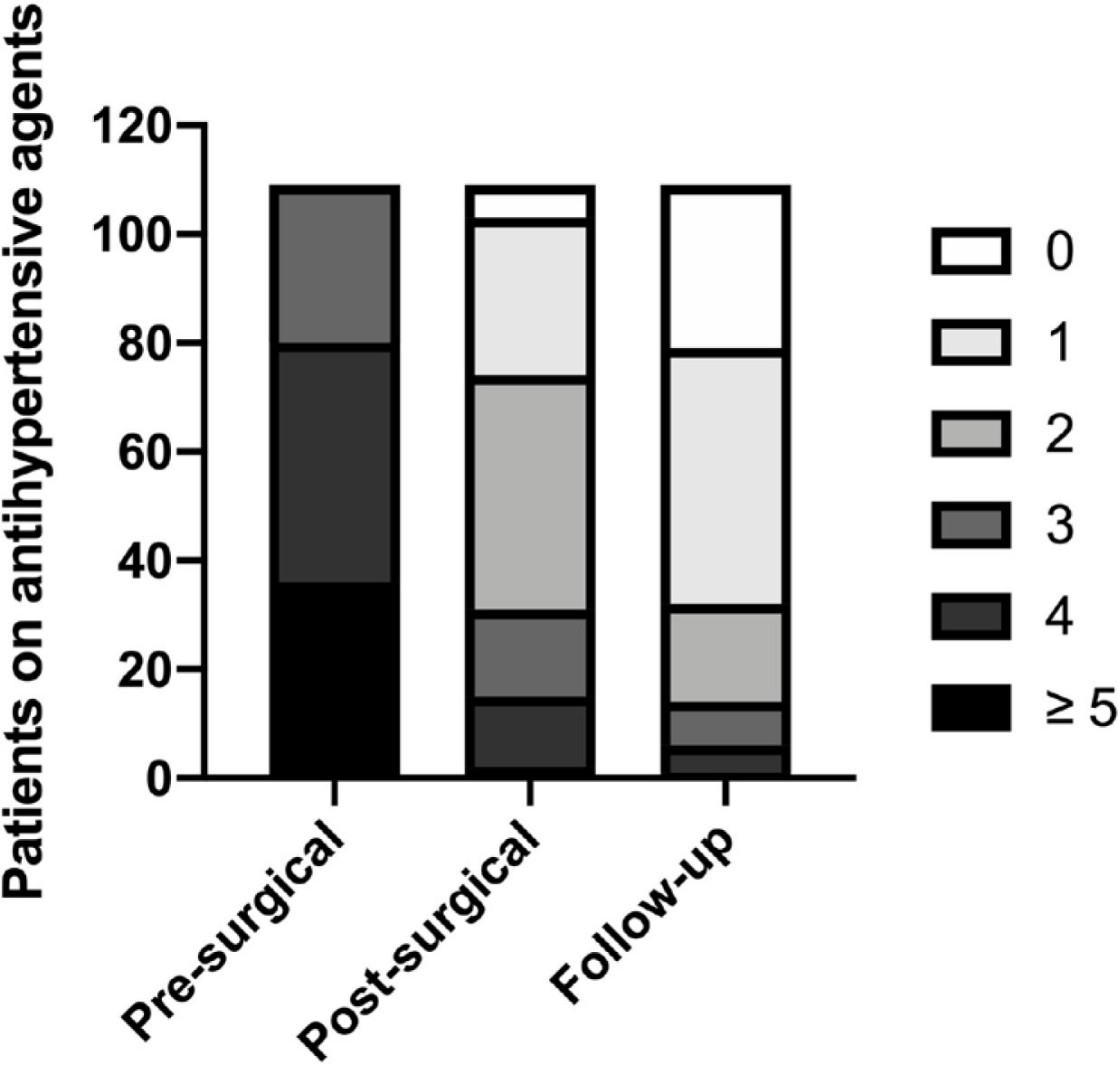
**Reduction in antihypertensive agents. Distribution of patients with the number of antihypertensive medications taking**.

**Figure 2:**
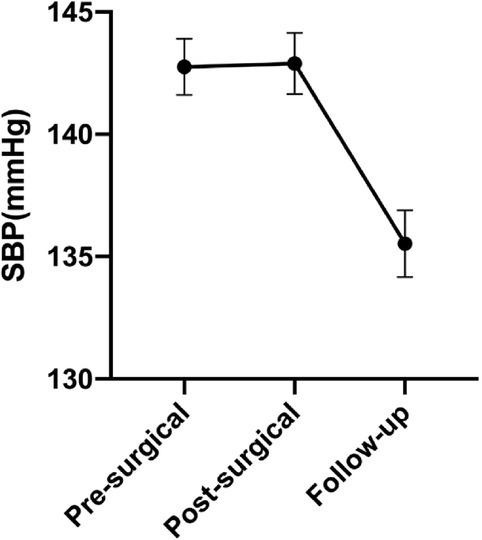
Blood pressure outcomes. SBP at pre-operative, post-operative and follow-up. SBP: systolic blood pressure.

Eight patients were lost in follow-up and no significant differences were found in their basal characteristics compared with the total participants. In 109 (93.2%) patients connected in the final follow-up, the mean follow-up duration was 36.2 months. In terms of BP, 27 (25 %) patients acquired complete normalization (BP < 140/90 mmHg without any aid of hypertensive drugs), 68 (62%) obtained improvement (similar or improved BP on fewer antihypertensive medications), while 14 (13%) were allocated as “non-response” (unaffected BP level on similar or more antihypertensive agents). In addition, on taking anti-hypertensive medications decreased from presurgical 4.24 to 1.21 at follow up (*P* < 0.01) (Figure 1). The amelioration in systolic blood pressure (SBP) is 7.2 mmHg, from 142.7 mmHg at baseline to 135.5 mmHg at follow-up (*P* < 0.01) (Figure 2).

### Image macro-adenoma and hypokalemia predict for postoperative BP normalization

Results of single factor analysis indicated several variables associated with postoperative BP normalization (Table 2), which includes image subtypes, image macro-adenoma, stroke history, plasma potassium, total cholesterol, low density lipoprotein (LDL) (All *P* < 0.05). Factors including age, sex, body mass index (BMI), preoperative SBP and DBP, anti-hypertensive classes, surgery type (total or partial adrenalectomy), hypertension onset age, hypertension duration, diabetes history, family hypertension history, smoking history, drinking history, creatinine, blood urea nitrogen, glomerular filtration rate, high density lipoprotein (HDL) and triacylglycerol (TG) were not found to have any evident correlation with the postoperative BP prognosis (All *P* > 0.05, results not shown here). Of note, plasma cortisol, adrenocorticotrophic hormone (ACTH), renin, aldosterone and aldosterone renin ratio were not found to have any evident correlation with postoperative BP prognosis. However, due to the unsuccessful cease of all interfering drugs before endocrine examination, predicative effect of endocrine factors needs further confirmation. *In patients with unilateral macro-adenoma (*N* = 34), tumor size was not statistically associated with postoperative BP normalization. Besides, no evident difference was observed in the follow-up durations between patients who achieved BP normalization or who didn’t (*P* = 0.476), which may suggest that BP outcomes after surgery did not swing much over time.

**Table 2:** Univariate associated with postoperative BP normalization

	Normalization (*n* = 27)	Non-normalization (*n* = 82)	*χ^2^*/t	*P* value
Image subtypes			6.220	0.014
Unilateral macro-adenoma	15 (44%)	19 (56%)		
Unilateral Combined lesions	6 (33.3%)	12 (66.7%)		
Unilateral adrenal hyperplasia	3 (10.3%)	26 (89.7%)		
Bilateral lesions	3 (10.7%)	25 (89.3%)		
Image macro-adenoma?			23.097	0.000
Yes	17 (56.7%)	13 (43.3%)		
No	10 (12.7%)	69 (75.3%)		
Stroke History	2 (7%)	21 (26%)	4.121	0.045
Plasma Potassium	2.74 ± 1.00	3.58 ± 0.89	16.992	0.000
Total cholesterol	4.33 ± 0.94	5.23 ± 1.12	8.117	0.006
LDL	2.58 ± 0.77	3.17 ± 0.88	5.685	0.020

LDL: low density lipoprotein; BP: blood pressure.

**Figure 3:**
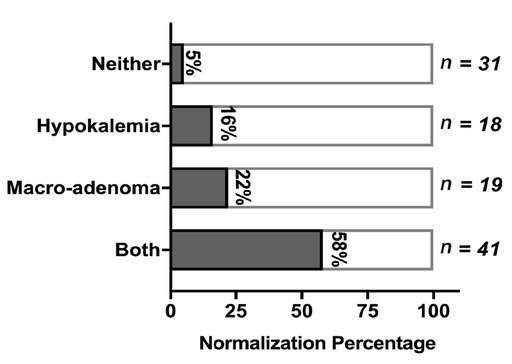
BP outcome in patients with both image macro-adenoma and hypokalemia. Postoperative hypertension normalization rate based on with or without image macro-adenoma and hypokalemia.

To exclude correlation effect between confounding factors, bivariate correlation analysis was conducted among factors owning evident association with BP prognosis, strong correlations were observed in three pairs of factors: total cholesterol and low-density lipoprotein (*r* = 0.939, *P* = 0.000), image subtypes and image macro-adenoma (*r* = 0.611, *P* = 0.000), image macro-adenoma and plasma potassium (*r* = 0.272, *P* = 0.004). Based on the above results and clinical meaning, we defined hypokalemia as plasma potassium lower than 3.5 mmol/L and hypercholesteremia as total cholesterol higher than 5.7 mmol/L. We enrolled image macro-adenoma, stroke history, hypokalemia and hypercholesteremia to perform a logistic regression analysis. As the results show (Table 3), image macro-adenoma and hypokalemia remains two independent predictors for postoperative BP prognosis. Hypertension normalization achieved in 58% patients who both manifested image macro-adenoma and hypokalemia history, far higher than the normalization rate in patients manifested only image macro-adenoma or hypokalemia, or none of the two features (*χ^2^* = 28.032, *P* = 0.000) (Figure 3).

**Table 3:** Image macro-adenoma and hypokalemia remains two independent predictors for post-operative BP normalization

	OR (95% CI)	*P* value
Image macro-adenoma		
No	1.00	
Yes	8.71 (2.67–28.37)	0.000
Hypokalemia		
No	1.00	
Yes	3.30 (1.03–10.63)	0.046

CI: confidence interval.

**Figure 4:**
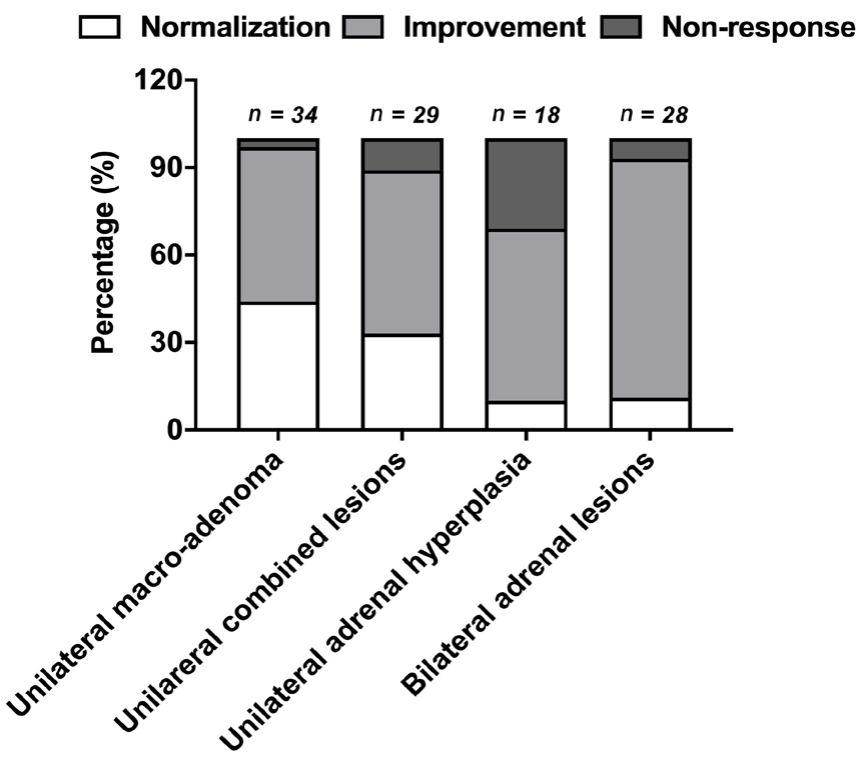
BP outcome based on image subtypes. BP outcomes in subgroup analysis based on image subtypes.

As mentioned before, image macro-adenoma is the strongest predictors for post-adrenalectomy BP prognosis in trHTN patients. We defined image macro-adenoma as the existence of unilateral macro-adenoma along with or without ipsilateral adrenal hyperplasia. As the subgroup analysis based on image subtypes shown in Figure 4, the BP outcome of patients with unilateral combined lesions is similar to patients with unilateral solitary macro-adenoma. However, in patients with unilateral adrenal hyperplasia, the BP normalization rate is remarkably poorer (10%), at the same level within patients with bilateral adrenal lesions (11%), much lower than that in patients with image macro-adenoma (*P* < 0.05). Besides, in the baseline characteristic comparison, for patients with unilateral adrenal hyperplasia, the hypertension onset age in patients was younger (33.0 *vs*. 40.3, *P* = 0.005), incidence of hypokalemia was lower (24% *vs*. 65%, *P* = 0.001), compared with patients with image macro-adenoma. All above implies that unilateral macro-adenoma, no matter if it coexisted with ipsilateral adrenal hyperplasia or not, is a strong predictor for BP normalization by adrenalectomy.

### Relatively low incidence of adverse events after surgery

Finally, we summarized postoperative adverse events in the level of different image subtypes (Table 4). Three patients underwent postoperative adrenal incompetence and required long-term glucocorticoid replacement (hydrocortisone) treatment; Hypokalemia was resolved in 48/50 (96%) patients, except in one patient with unilateral adrenal hyperplasia and the other patients with bilateral adrenal lesions; adrenal lesions recurrence happened in one patients with unilateral adrenal hyperplasia; new onset stroke occurred in one patients with unilateral hyperplasia and the other three patients with bilateral adrenal lesions, which may partly result from their poor BP outcomes. In summary, the incidence of postoperative adverse events was small.

**Table 4:** Postoperative negative events

	Unilateral macro- adenoma (*n* = 34)	Unilateral combined lesions (*n* = 18)	Unilateral adrenal hyperplasia (*n* = 29)	Bilateral adrenal lesions (*n* = 28)
Glucocorticoid replacement	2	1	0	0
Reoccurred hypokalemia	0/22	0/9	1/7	1/12
New stroke	0	0	1	3
Recurrence	0	0	1	0

## Discussion

Our current study, for the first time, investigated the longterm BP outcome of adrenalectomy within trHTN patients. In 109 participants who were successfully contacted in the last interview, BP of 27/109 (25%) patients obtained complete normalization and 68/109 (62%) obtained improvement. On taking antihypertensive agents, numbers decreased from preoperative 4.24 to 1.21 at follow-up, along with 7.2 mmHg reduction in SBP. Image macro-adenoma and hypokalemia were found to be the two strongest predictors for postoperative BP normalization. In addition, few postoperative adverse complications were observed. Herein, we believe our result suggests adrenalectomy is effective and safe in the BP controlling within trHTN patients and it could be recommended as a novel strategy in trHTN management.

The discovery of adrenal lesions in the vast majority of our participants was incidental and its relationship with their uncontrolled BP remains elusive. Unexpectedly, our participants acquired a huge alleviation in BP after surgical removal of abnormal adrenal. To our knowledge, functional adrenal lesions including aldosterone-producing adenoma (APA),^[12]^ adrenal Cushing's syndrome^[13]^ and pheochromocytoma constitute the most frequent reasons of secondary hypertension.^[21]^ Overproduced adrenal hormones up-regulate water-sodium retention or sympathetic system activation was believed to be the underlying culprit in adrenal lesions. In addition, recent studies on NFATs induced deeper prospection into the role of adrenal lesions in hypertension. For example, Li *et al*.^[22]^ reported 49.1% of the NFATs patients had a better control of their blood pressure level after adrenalectomy. Another study conducted in 77 surgically treated NFATs patients, found 27 (35%) patients cured and 26 (31%) improved their BP after surgery.^[23]^ Several studies confirmed that NFATs cohorts suffered an underestimated prevalence of metabolic problems, including hypertension, diabetes mellitus and obesity.^[24, 25, 26]^ Our results, in line with the aforementioned studies, collectively suggest the unprecedented intimate correlation between hypertension and adrenal lesions, alone with or without endocrine disorders. Interrogating into correlation between BP and adrenal lesions and the application of minimally invasive adrenalectomy in BP management deserves more clinical attention.

In addition, image macro-adenoma and hypokalemia were found to be two independent predictors for postoperative BP normalization, which is believed to be consistent with the ground-breaking finding in the primary aldosteronism made by Küpers *et al*.^[27]^ They reported that adrenal venous sampling (AVS) lateralization could be accurately predicted by the combination of a unilateral adrenal mass of exceed 10 mm on image, plus hypokalemia, or effective glomerular filtration rate more than 100 ml/min, thus proceed directly to laparoscopic adrenalectomy. These results implicate a mix of patients with functional adrenal disorders in our set of participants.^[28, 29, 30]^

The biggest flaw in our current study is the lack of accurate endocrinal examinations data. Complete discontinuation of all interfering before endocrinal examinations in our participants are confined by high cardiovascular safety concerns. If ideally, every adrenal lesion should be given an accurate biochemical diagnosis, nonetheless, our results are more likely to reflect a “real world” scenario; part of patients with adrenal lesions cannot get accurate biochemical diagnosis. Herein, our study aims at providing clinical reference to this population who are trHTN patients without definite adrenal lesions.

In general, our study is innovative and no similar studies have been reported before. We stated the efficacious and safe effect of adrenalectomy on BP trimming in trHTN patients and found out image macro-adenoma and hypokalemia correlated with favorable postoperative BP prognosis. We suggest adrenalectomy could be considered as a BP controlling strategy in trHTN, since it creates great BP profits and low adverse events incidence. This study may make a further progress in the clinical management and outcomes in trHTN patients.
